# Prevalence of Depression among Medical Students of a Tertiary Care Teaching Hospital

**DOI:** 10.31729/jnma.4738

**Published:** 2019-12-31

**Authors:** Nikhil Shrestha, Neharika Shrestha, Smriti Khanal, Sujata Dahal, Roshan Lama, Prashant Simkhada, Sudarshan N Pradhan

**Affiliations:** 1 Oxford University Clinical Research Unit-Nepal, Lalitpur, Nepal; 2 Tokha Chandeshwori Primary Health Center, Kathmandu, Nepal; 3 Jibjibe Rural Hospital, Rasuwa, Nepal; 4 Deurali Primary Health Center, Nuwakot, Nepal; 5 Kanti Children's Hospital, Kathmandu, Nepal; 6 Kathmandu Medical College, Sinamangal, Nepal

**Keywords:** *depression*, *medical students*, *prevalence*

## Abstract

**Introduction:**

Depression is one of the major problems encountered by medical students. This may cause a negative effect on cognitive functioning and learning of students resulting in poor health care and service delivery in future. The presence of this major problem is necessary to be evaluated. Enough recent data regarding its prevalence is not available in our country. Thus, the main aim of this study is to find the prevalence of depression among medical students of a tertiary care teaching hospital.

**Methods:**

This is a descriptive cross-sectional study was conducted among medical students of a tertiary care teaching hospital over a four-month period (July to October 2019) among undergraduate medical students. Ethical clearance was received from the Institutional Review Committee of the same tertiary teaching hospital. Random sampling technique was used to collect data to meet the calculated sample size. Data analysis was done in the Statistical Package for Social Sciences. Point estimate at 95% Confidence Interval was calculated along with frequency and proportion for binary data.

**Results:**

The prevalence of depression among selected medical students of Kathmandu Medical College and Teaching Hospital is 59 (27.2%) at 95% Confidence Interval (21.28-33.12%). Thirty (14%) of the participants were mildly depressed, 21 (10%) moderately depressed while 8 (4%) were severely depressed according to Beck's Depression Index II.

**Conclusions:**

Prevalence of depression among medical students is relatively significant as found in similar studies done in other centres. Thus appropriate programs and strategies should be implemented to avoid depression from causing a negative effect on cognitive functioning and learning of students and create a favorable environment for students to talk about their mental health issues.

## INTRODUCTION

Medical students undergo many stressful situations. They have to deal with substantial academic, psychological as well as existential stressors during the duration of their undergraduate years.^1^ Studies have thus shown that medical students experience depression at a much higher rate than that of the general population,^2,3^ this has been an important issue in both the developed as well as developing nations.

The future doctors themselves are at risk of being victimized by depression. Over the course of their medical training, their mental health tends to deteriorate even more.^4-9^ This has lead to students opting for potentially detrimental coping mechanisms such as excessive use of alcohol, and the use of other substances.^10^ Depression has even been associated with suicidal ideation.^7,9,11,12^ Furthermore, in spite of their training medical students are unsuccessful to comprehend depression as a major illness, that necessitates proper medical treatment when it comes to caring for themselves.^14^

There has been limited study in the past, thus this study aims to find the prevalence of depression among medical students of Kathmandu Medical College Teaching Hospital, a tertiary care centre.

## METHODS

A descriptive cross-sectional study was conducted in a tertiary hospital, KMCTH in Nepal. After taking the ethical clearance from the Institutional Review Committee of Kathmandu Medical College and Teaching Hospital, data was collected from 217 medical students of Kathmandu Medical College Teaching Hospital from July to October 2019. The sample size of this study was calculated using the formula,

Sample size was calculated using the formula;

$$\begin{array}{ll} \text{n} & ={\text{Z}}^{\text{2}}\times \text{(p}\times \text{q)}/{\text{e}}^{\text{2}}\\ & =\text{1}{\text{.96}}^{\text{2}}\times \text{0.5}\times \text{(1}-\text{0.5) / 0}{\text{.05}}^{\text{2}}\\ & =384 \end{array}$$

where,

n= required sample size
p= prevalence of depression among medical students (50%)
q= 1-p
e= margin of error, 5%
Z= 1.96 at 95 % CI

In the teaching hospital, population(N): 450

Adjusted Sample size= n/ 1+n-1/N

                  = 207

Therefore, the calculated sample size was 207. Adding the 5% non-response rate, the sample size that was taken is 217.

Simple random sampling technique was applied. The list of the students in each batch was taken from the hospital's administration office, a consecutive number was given to the list. A random number table was utilized to select the sample. Medical students enrolled currently in the MBBS curriculum in the tertiary teaching hospital who gave consent were included in the study. Students in their internship year, those chronically absent or seriously ill at the time of the study were not included in the study. Randomly selected students were informed about the study and those willing to participate were given consent paper for signature. Then a self-administered validated Beck's Depression Inventory(BDI)-II questionnaire^13^ was distributed and collected from participants. Despite simple random sampling, the following bias could occur such as; Information bias, Reporting bias, Social desirability bias and Non-response bias.

Data collected was thoroughly checked for its completeness. Only those completed forms were included in the study. The data was then coded and double-entry done in IBM Statistical Package for Social Sciences version 23.0. The data was processed and analyzed by using simple descriptive statistics; in terms of percentage and frequency.

## RESULTS

The period prevalence of depression among medical students in Kathmandu Medical College Teaching Hospital is 59 (27.20%) 95% Confidence Interval (21.28-33.12%). Thirty (14%) of the participants were mildly depressed, 21 (10%) moderately depressed while 8 (4%) were severely depressed according to Beck's Depression Index II.

**Figure 1:**
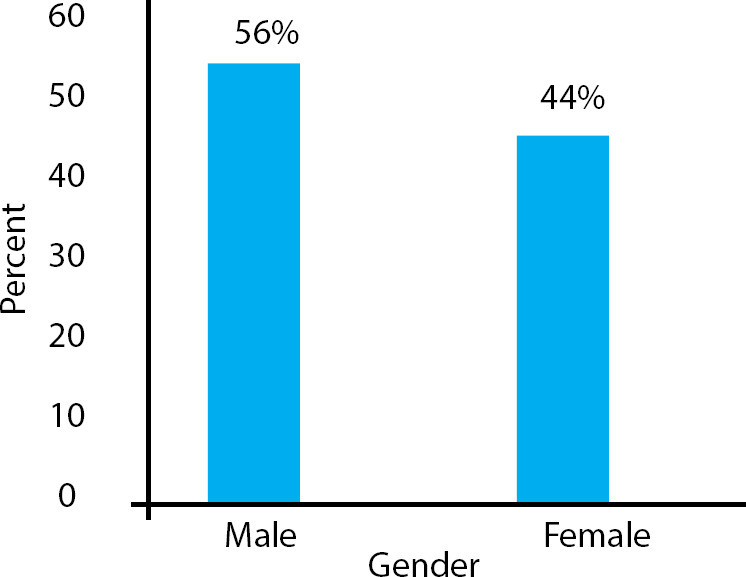
Gender wise distribution of participants.

**Figure 2:**
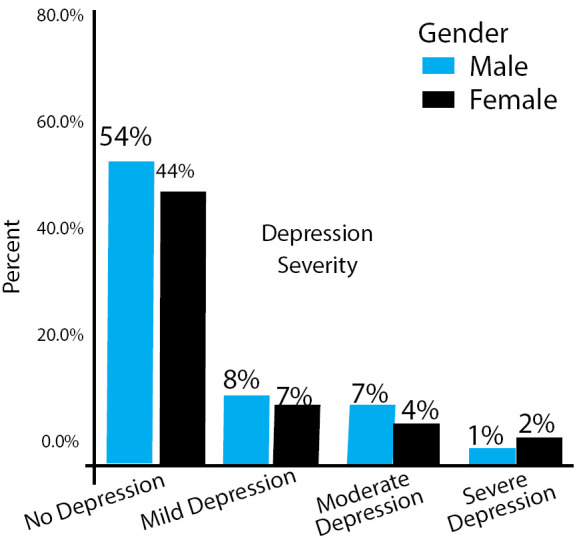
Gender wise prevalence of depression.

Among 217 participants, there were 121 (56%) male and 96 (44%) female; depression was more prevalentamong females than males (Figure 1). Fourteen (7%) of female participants were mildly depressed, 9 (4%) moderately depressed and 5 (2%) severely depressed. Similarly, 16 (7%) of male participants were mildly depressed, 12 (6%) moderately depressed and 3 (1%) severely depressed (Figure 2).

**Figure 3:**
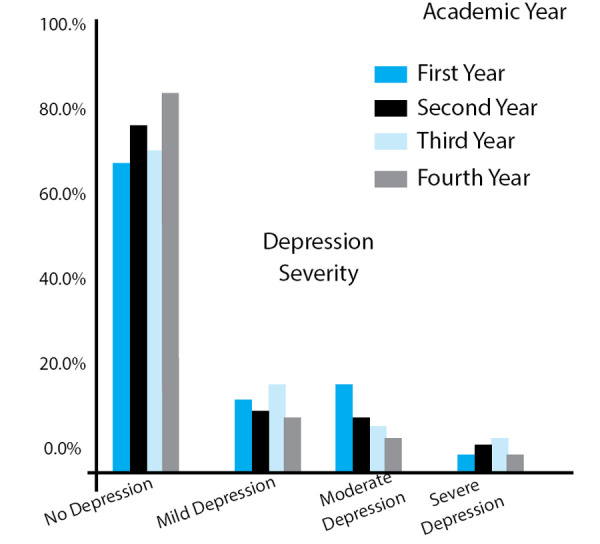
Year wise distribution of Depression.

As medical students progressed to their clinical years, the prevalence of depression tends to increase. The prevalence of depression in 1st year, 2nd year, 3rd year and final year students was 14%, 23%, 30%, and 14% respectively. This gave a prevalence of 34% in preclinical students in contrast to 44% in clinical students (Figure 3).

Regarding socio-demographics, almost all of the participants were within the age-group of 18-29 years. And none of the participants was married. Depression was more prevalent among females 28 (29%) compared to males 31 (26%).

Now moving to other factors, among the 27% of depressed participants, only 2 (1%) had a family history of depression while 57 (26%) of the depressed participant had no such family history. In this study, we also observed that more participants who were depressed used smoking or alcohol to cope with stress (46%) compared to non-depressed participants (29%).

Finally, we also observed that 23 (11%) of the participant who felt like they were academically incompetent were depressed.

## DISCUSSION

Due to substantial academic, psychological as well as existential stressors that the medical students should cope with,^1^ these subgroup populations are at higher risk of experiencing psychological and mental problems such as depression.

In our study, the prevalence of depression is found to be 27.20% which as compared to the previous study is lower. In the study done by Basnet B, et al.^15^ the prevalence of depression was 29.7% with prevalence among female students being higher than those in males, 32% in females and 28% in males, which was similar to our study. This finding is also supported by a similar study done in Bristol by Knipe et al. which found a prevalence of 27% in females and 19% in males.^16^

A study done by Adhikari A, et al. found a similar prevalence of 29.2%, in their study found that the prevalence of depression was higher among preclinical students (33%) compared to clinical students (22%),^17^ which is in contrast to our study. However, a systematic review that was done by Rotenstein L et al. at Harvard found no such difference.^18^ But a similar study done by Sreeramareddy C, et al. found that the prevalence rate was higher among clinical science students.^19^ 15%, 18.9% and 24% in third, fourth and final year students respectively which was similar to our study which found that prevalence tended to increase as students started their clinical years (i.e. third year). Juggling time between postings, theory classes and trying to gain clinical experience can be the reason for overwhelming the students more at the beginning of clinical years, which could be the reason for the high prevalence in third-year students.

In comparison to students of other faculty, medical students have more stigma when it comes to mental health issues since their competency is also questioned,^20^ leading to more students having difficulty talking about their mental health problems. This in addition to academic stress and expectation from family might have the reason for higher percent depression (26%) without any predisposing factor-like family history (1%).

The limitation of our study is that since the study was carried out only in a single teaching hospital, the findings of the study cannot be generalized to all student populations. During the time of the study, some student groups were in their exam period which could influence the responses resulting in an overestimation of the frequency of symptoms. This study being a cross-sectional study, our results only portray a point estimate, we are unable to determine if the proportion of students having sustained symptoms.

## CONCLUSIONS

The prevalence of depression among medical students was found to be similar to the other similar studies done in other centres. The prevalence of depression among medical students is higher compared to the general population. This suggests the need of confidential and accessible mental healthcare and investment of resources into developing strategies and programs which reduce stress such as workshops on topics such as depression & suicide, anxiety, burnout, and stress management decreasing the stigma related to it, along with trainings about counseling for mental health issues as a curriculum.
